# A divide-and-conquer strategy in tumor sampling enhances detection of intratumor heterogeneity in routine pathology: A modeling approach in clear cell renal cell carcinoma

**DOI:** 10.12688/f1000research.8196.2

**Published:** 2016-04-25

**Authors:** José I. Lopez, Jesús M. Cortes

**Affiliations:** 1 Department of Pathology, Cruces University Hospital, Biocruces Research Institute, University of the Basque Country (UPV/EHU), Barakaldo, Spain; 2 Quantitative Biomedicine Unit, Biocruces Research Institute, Barakaldo, Spain; 3 Ikerbasque: The Basque Foundation for Science, Bilbao, Spain; 4 Department of Cell Biology and Histology, University of the Basque Country (UPV/EHU), Leioa, Spain

**Keywords:** Intratumor heterogeneity, clear cell renal cell carcinoma, pathologist, tumor sampling, divide-and-conquer strategy, computational modelling, laboratory costs

## Abstract

Intratumor heterogeneity (ITH) is an inherent process in cancer development which follows for most of the cases a branched pattern of evolution, with different cell clones evolving independently in space and time across different areas of the same tumor. The determination of ITH (in both spatial and temporal domains) is nowadays critical to enhance patient treatment and prognosis. Clear cell renal cell carcinoma (CCRCC) provides a good example of ITH. Sometimes the tumor is too big to be totally analyzed for ITH detection and pathologists decide which parts must be sampled for the analysis. For such a purpose, pathologists follow internationally accepted protocols. In light of the latest findings, however, current sampling protocols seem to be insufficient for detecting ITH with significant reliability. The arrival of new targeted therapies, some of them providing promising alternatives to improve patient survival, pushes the pathologist to obtain a truly representative sampling of tumor diversity in routine practice. How large this sampling must be and how this must be performed are unanswered questions so far.  Here we present a very simple method for tumor sampling that enhances ITH detection without increasing costs. This method follows a divide-and-conquer (DAC) strategy, that is, rather than sampling a small number of large-size tumor-pieces as the routine protocol (RP) advises, we suggest sampling many small-size pieces along the tumor. We performed a computational modeling approach to show that the usefulness of the DAC strategy is twofold: first, we show that DAC outperforms RP with similar laboratory costs, and second, DAC is capable of performing similar to total tumor sampling (TTS) but, very remarkably, at a much lower cost. We thus provide new light to push forward a shift in the paradigm about how pathologists should sample tumors for achieving efficient ITH detection.

## Introduction

Neoplasia is the result of multiple and complex disturbances of the cellular metabolism ^1, 2^. Although the arrival of sophisticated technological devices like massive sequencing has improved the knowledge on the molecular mechanisms underlying carcinogenesis, intratumor heterogeneity (ITH) still remains poorly understood ^3^. ITH, the fact that a given tumor is intrinsically diverse across different regions, is of crucial importance for both basic and clinical researchers. Whilst basic researchers focus on ITH because it reflects the complexity of tumor development, clinical researchers do it because ITH is a major obstacle for the success of new patient therapies. ITH develops stochastically in both time and space domains, in a manner that the resulting ITH patterns are unique and utterly unpredictable. Modern pathologists have the challenge to determine ITH efficiently, helping basic researchers in the detection of mutational signatures and oncologists in the selection of better personalized therapies. This paper shows an affordable, very simple but efficient method to improve ITH detection in clear cell renal cell carcinoma (CCRCC) in the routine practice without increasing costs. The method we suggest is based on the Divide And Conquer (DAC) strategy ^4^, which consists in recursively breaking down a given problem into simpler parts (divide) until they are simple enough to be efficiently solved (conquer). Our approach, that can be applied to any other tumor type, is supported by a computational modeling approach.

## The context

Renal cancer, a common neoplasm in Western Countries with more than 62,000 expected deaths in the USA in 2016 ^5^, is a complex disease with several distinct tumor subtypes ^6, 7^. CCRCC is by far the most common renal cancer histological variant, accounting for 75 to 80% of renal neoplasms in adults ^8^. CCRCC is a clinically aggressive neoplasm, where radical surgery is the only treatment with significant impact on patient survival ^9^. Chemo- and radiotherapy are inefficient so far and modern targeted therapies obtain only partial results ^10^. This context makes CCRCC one of the most attractive challenges in cancer research for the coming years. As a result, many investment efforts are being made worldwide to improve CCRCC response to new therapies.

**Figure 1.**
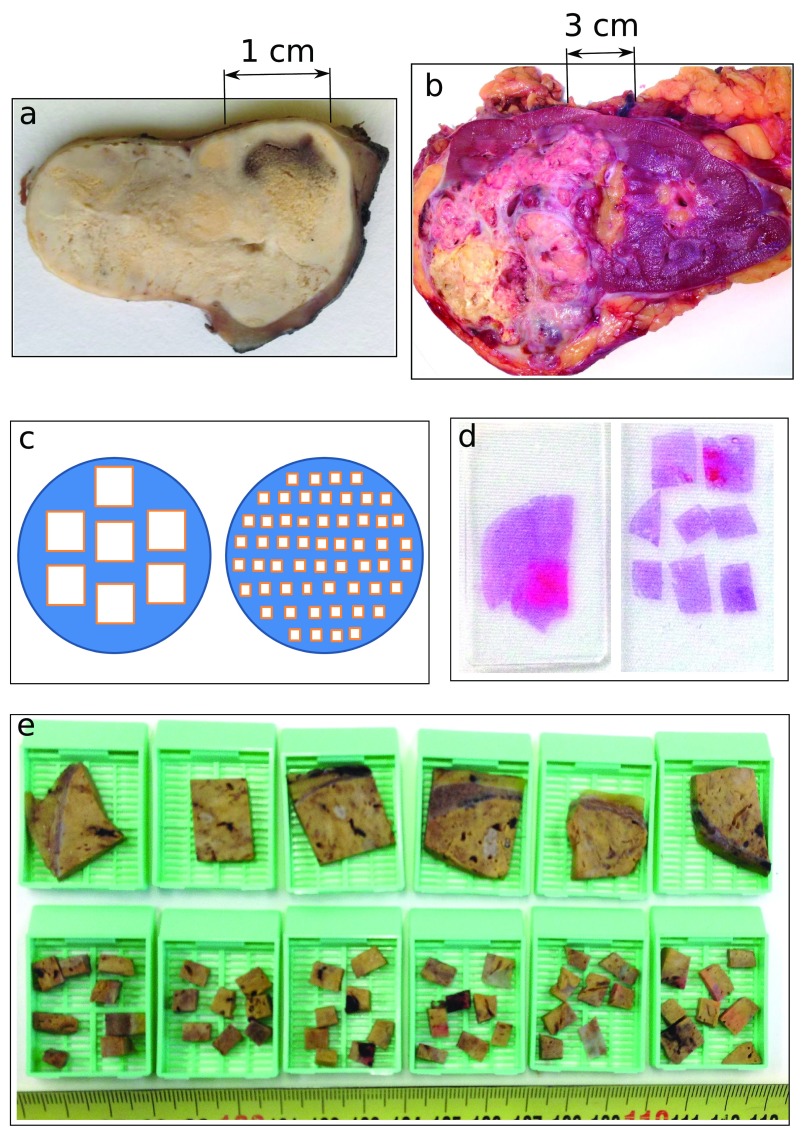
ITH in CCRCCs may be hidden ( **a**) or evident ( **b**) to the naked eye during the management of surgical specimens, an issue that is critical for subsequent tumor sampling. The RP strategy selects for the analysis 1 sample per cm of tumor diameter, as reflected at the left side of panels **c**– **d** ( **c**, diagram; **d**, histological slide) and at the top row of blocks in panel **e**. In contrast, the DAC strategy (the alternative we are suggesting here) selects more small-pieces for tumor sampling (for instance, panels **c**– **e** show how while RP selects big blocks, DAC selects 8 small-pieces per each large block) but the pieces are randomly chosen along the tumor. Importantly, both methods RP and DAC demand the same laboratory costs.

CCRCC is a paradigm of a heterogeneous tumor from several viewpoints ^11– 18^, where not only different CCRCCs are distinct from each other, but the ITH within the same CCRCC is also high. CCRCC presents ITH patterns which vary from obvious to subtle depending on very different approaches. In some cases, for instance, ITH is evident to the naked eye, while in others it is not ( Figures 1a & 1b). Although some CCRCCs might seem apparently homogeneous at first sight, they can actually be heterogeneous under the microscope. It is also possible that other CCRCCs that appear homogeneous at the microscope are in fact heterogeneous at the molecular level, where different gene mutations (each one pursuing a different clinical outcome) can be present at different regions, i.e., gene mutations located at the *BAP-1* gene are associated to high-risk of aggressive clinical outcome; mutations at the mTOR pathway make the tumor sensitive to targeted therapies; mutations at the *PBRM1* gene are linked to low-risk clinical aggressiveness ^15, 19^. In contrast, some mutations like those located at the *VHL* gene seem to be truncal (early) and generalized for all CCRCC regions ^15^, which makes the *VHL* mutations potential targets for modern therapies; but unfortunately, all the assays performed to date to target this pathway have obtained disappointing results ^20^. Importantly, this therapeutic failure has been associated to ITH; and in particular, to the branching evolution patterns that malignant cells follow across different regions ^11, 14– 16^. This unpredictable regional variability makes impossible to date to design efficient therapeutic strategies for these neoplasms. This fact explains the high 5-year cancer-related mortality of CCRCC, currently reaching up to 40% ^21^.

The expected effect of modern targeted therapies is tumor necrosis, which is achieved by acting against either specific gene mutations or abnormal protein products generated by neoplasia ^22^. Depending on the response to these drugs, CCRCCs are divided into responders to therapy and non-responders, and this makes the difference between having success or failure in the clinical setting. After the CCRCC has been surgically removed, responders show generalized tumor necrosis and are associated with longer survival rates, whilst non-responders typically display a mixture of tumor necrosis and a viable tumor, and are associated with shorter survivals.

## The problem

Pathologists are the clinicians who handle surgical specimens and decide which parts of the tumor will be studied and which others won’t. If tumor size is small (≤3 cm in diameter), pathologists can analyse the entire tumor. However, CCRCCs are usually much larger than that, reaching up to 10–15 cm in tumor-diameter or even more, and this fact makes the sampling of the entire tumor not cost-effective. For this reason, pathologists perform tumor sampling following internationally accepted protocols ^23– 25^, a selection made with the intention to achieve obtained samples which are good representatives of the entire tumor. In particular, the accepted consensus is to obtain 1-centimetre-in-length sample per 1-centimetre-of-tumor-size plus additional similar samples from every suspicious region detected by the naked eye. ITH, however, is frequently hidden in apparently identical areas, as we have previously mentioned, and this is what makes ITH an important limitation to the performance of these protocols. A second limitation is related to the percentage of total tumor sampled, in cases where tumor size is larger than 3 cm in diameter the current practice leaves out the analysis of a very significant portion of the tumor (for instance, more than 95% of a 10 cm-in-diameter CCRCC is not included for analysis).

Once the sampling for diagnosis is performed, the remainder of the tumor is first stored and then destroyed, so that, the amount of crucial information forever lost remains largely unknown. Previous studies on total samplings assays performed in two short series have shown very concerning data ^12, 13^, and in particular, that the detection of highly aggressive areas (associated to high ITH) are missed after conventional samplings, which is not acceptable in modern clinical practice. Being aware of the limitations provided by current protocols in unveiling ITH, both clinicians involved in patient treatment and basic researchers are appealing for urgent solutions ^26^. However, pathologists have not provided a well sustained solution to this problem so far, and the latest updates on sampling protocols ^24, 25^ apparently are not taking this problem into consideration. To overcome these limitations, several authors have recently developed algorithms to quantify ITH when very limited tissue is available for analysis ^27– 30^.

The appropriate selection of tumor samples falls absolutely into the pathologist’s responsibility. Importantly, a deficient or incomplete tumor sampling will give rise to a deficient or incomplete histological and/or molecular study, and this deficiency may have important consequences for patient treatment. It is indeed a paradox that such crucial information affecting the patient and obtained by using such sophisticated and expensive high-tech devices do drastically depend on the tumor samples that were selected based on dogmatic rules.

Despite these limitations, the classical pathologic routine morphology under the microscope still remains a decisive source of information for ITH discovery. Indeed, Andor *et al.* ^31^ showed very recently that the nuclear and cellular morphology associated with tumor aggressiveness observed in histological sections correlate with genetic ITH in several tumor types, renal cancer included.

## A solution

The solution for ITH detection must be affordable and workable at the same time, balancing scientific accuracy and cost. A total tumor sampling (TTS), although an ideal solution, is utterly unaffordable for most of the CCRCCs, as the huge number of samples generated would collapse the laboratory workflow. But at the other extreme, the current routine protocol has proven to be insufficient. Once arrived at this point, the key question is as follows: How can pathologists overcome this deficiency? An increase in the number of random samples would obviously increase the probability of finding the hidden ITH, as it has been empirically proposed recently ^15, 18, 26^, but, how large such an increase should be? Although (as far as we know) there is no answer to these questions at this time, it is possible to increase the number of samples to some degree while keeping the same cost fixed. We propose here a solution that is based on the Divide And Conquer (DAC) strategy. Thus, if we assume that the tumor has a regular shape (e.g., a circle in Figure 1c), straightforward reasoning says that 56 small samples placed in 7 blocks might have a higher chance for detection of ITH as compared to 7 large samples placed in the same number of blocks ( Figures 1c–1e). These figures show that more regions can be assessed without increasing laboratory costs (pathology laboratory costs are mostly based on the number of paraffin blocks used for the analysis, where the higher the number of blocks, the higher the cost).

## Modeling approach

For simplicity reasons, tumor shape was modeled using a 2D square of side L. ITH was represented by the *γ* matrix, defined by the elements:

$${\left(\gamma \right)}_{ij}\equiv \left\{0,1,2,3,\ldots ,C\right\}\;(\text{Eq}.1)$$

with *i*, *j* = 1, ..., *L* and where *i* indicates row and *j* column. The zero value models homogeneity, rather non-zero values model the presence of ITH at the tumor position given by ( *i*, *j*). The matrix *γ* can account for *C* (in principle, an arbitrary number) different ITH types.

**Figure 2.**
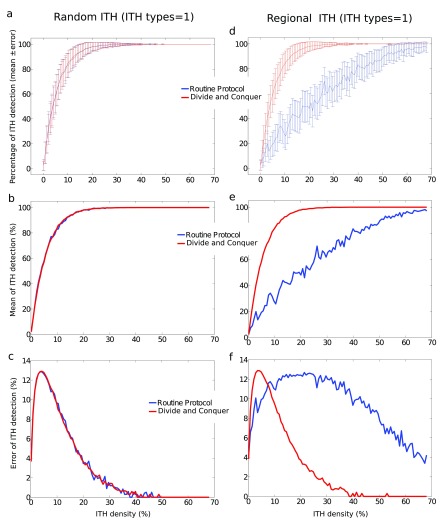
Results from the computational modeling approach show that, for tumors with ITH types=1, DAC (red) performs similar to RP (blue) for random ITH ( **a**– **c**) but the former outperforms the latter for regional ITH ( **d**– **f**). **a**, **c**: Percentage of ITH detection (mean ± SD) as a function of the percentage of ITH density defining for each tumor. SD was calculated across *N* different repetitions of the same strategy and across *M* different tumors. **b**, **e**: similar to **a**, **c** but we only represented the mean. **c**, **f**: similar to **a**, **c**, but we only represented SD. Notice that, not only the mean of ITH detection was drastically enhanced by DAC, but SD was substantially smaller, meaning that DAC in comparison to RP is more efficient and more reliable (less variable). Exact values for all simulated parameters are given in the text.

**Figure 3.**
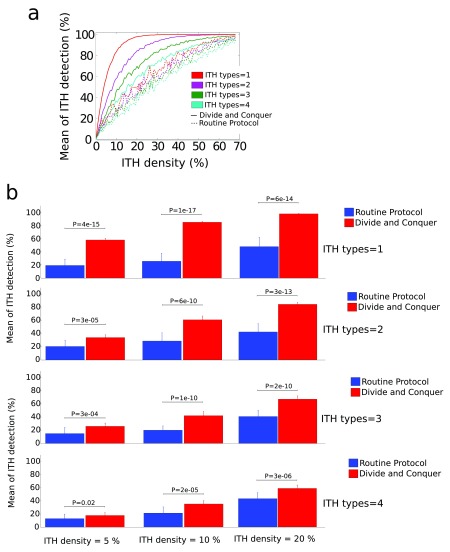
Results from the computational modeling for *C* = 4 ITH types and the situation of regional ITH. Similar to Figure 2 for *C* = 1, DAC performed equally as RP for random ITH (not visualised). **a**: regional ITH, DAC plotted as solid lines, RP as dashed ones. Different colors correspond to different ITH types. The mean of ITH detection (measured in %) is represented as function of the ITH density (also in %). Notice that, for the simulations we have performed here, DAC performed better in detecting all the *C* = 4 ITH types in comparison to the performance that RP achieved when only 1 ITH type existed. **b**: P-values after t-test (as implemented in Matlab, function *ttest*) showing significant differences in performance between DAC (blue) and RP (red) for different values of ITH density.

Two classes of ITH were simulated, random ITH (ranITH) and regional ITH (regITH), the latter being the situation more realistic with regard to how ITH typically is found in tumors ^11^. For both ranITH and regITH situations, ITH was simulated using an iterative method with *h* = 1, ..., *H* steps and where the initial condition was for all the cases *γ* = 0. At each step *h*, a 2D position ( *i*, *j*) is chosen at random and a value of ( *γ*) _*ij*_ = 1 *or* 2 *or* 3 *or*... *or* *C* is assigned with probability 1C. After the H steps, the *γ* matrix was fixed and defined the tumor ITH configuration to be detected by tumor sampling. The percentage of ITH density associated to a given tumor was defined as $\rho \equiv \frac{H}{L\times L}\times 100$ (c.f., x-axis in Figure 2 and Figure 3).

For the RanITH situation, the matrix elements of *γ* were randomly generated at position ( *i*, *j*), with no constraints for *i* and *j*, and this occurred for all the *H* steps. In contrast, for RegITH, only at the first iteration ( *h* = 1) the value of *γ* was assigned at position ( *i*, *j*) with no constraints for *i* and *j* (using the same procedure as for ranITH), but for the following iterations ( *h* ≥ 2), either the new chosen *i* or the new *j* was constrained to be necessarily a neighbor index of any of all the previously chosen *i* or *j*.

For a given tumor, and after introducing an ITH configuration defined by *γ* ( Equation 1), we repeated *N* times (and separately) two different strategies: routine protocol (RP, the one accepted in routine pathology) and our alternative, the DAC strategy. For each repetition and strategy, we calculated the number of successfully detected ITH sites for each of the *C* ITH types, i.e., detecting the value of ( *γ*) _*ij*_ = 1 for ITH subtype = 1, the value of ( *γ*) _*ij*_ = 2 for ITH subtype = 2, … , and the value of ( *γ*) _*ij*_ = *C* for ITH subtype = *C*. Results were averaged across the *N* repetitions and also across *M* different tumors (each one with a different ITH configuration).

For the two strategies, RP and DAC, the total number of blocks for each repetition was equal to *Q* (as explained above, this number is modeling the laboratory costs). For DAC, all the *Q* sites were chosen at random with no constraints for *i* and *j*. For RP, the first site, defined as ( *i*, *j*) ≡ ( *I*, *J*), was chosen at random with the constraint of *d* + 1 ≤ *I*, *J* ≤ *L* − *d*, where *d* is an index controlling the block size used for tumor sampling. For the following sites to be inspected (up to the number *Q*), we chose *i* = { *I* ± 1, *I* ± 2, *I* ± 3, ..., *I* ± *d*} and *j* = { *J* ± 1, *J* ± 2, *J* ± 3, ..., *J* ± *d*}, that is, *d* sites up to *I*, *d* sites down to *I*, *d* sites right to *J* and *d* sites left to *J*.

A generalization to 3D tumors is straightforward; notating tumor sites with triplets ( *i*, *j*, *k*) and defining for RP also the *d* sites backwards to *K* and the *d* sites forwards to *K*.

The code for the modeling approach was implemented in Matlab (The Mathworks, Inc, version 2012a) and is available at Code1, which internally calls to other functions, Code2– Code5.

Code1: Main Matlab function to start simulation. To run it in Matlab, simply run “simula2D.m”. Some comments in.

Code2: A small function “creates_random_ITH_cube2D.m” called from “simula2D.m”.

Code3: A small function “creates_regional_ITH_cube2D.m” called from “simula2D.m”.

Code4: A small function “RP2D.m” called from “simula2D.m”.

Code5: A small function “DAC2D.m” called from “simula2D.m”.

We model the possibility of having different ITH types within the same tumor. Results from the modeling approach are shown in Figure 2 (ITH types=1) and Figure 3 (ITH types=4). RP and DAC performed similar for ranITH ( Figure 2) and this happened independently on *C* (the number of ITH types). However, for regITH, DAC significantly outperformed RP for any *C* number ( Figure 2 and Figure 3). Other parameters for the simulations were: *L* = 27 (side of 2D square), *C* = 4 (ITH types), *H* (number of sites with ITH) varying from 1 to 500 (or equivalently the ITH density *ρ* varying from approx. zero to 68%), *N* = 500 (repetitions number for the two RP and DAC strategies), *M* = 15 (number of simulated tumors), *d* = 4 (block size), which is equivalently to *Q* = 17 (total number of blocks for ITH detection by applying both RP and DAC strategies).

Finally, it is important to remark that our approach makes use of random sampling for both RP and DAC strategies. Although we are aware that other strategies based on non-random sampling might be chosen, as for instance, by ranking possible states to be chosen by an Energy function which is state dependent, ie., Monte Carlo sampling ^32– 34^, the modeling approach here provides a first-stage solution, but extensions and improvements can be done in future work.

## Estimation of laboratory costs for the DAC strategy

We have shown based on a modeling approach that DAC strategy outperforms RP at the same cost as both strategies make use of the same number of blocks to search for ITH. But, what is the cost of the DAC strategy in relation to the cost of the TTS strategy? The answer to this question can be also estimated using a similar modeling approach as explained in the previous section. Before starting, it is interesting to remark that the TTS strategy is an ideal scenario for two reasons; first, TTS performance in detecting ITH is by definition 100% and second, TTS cost is the maximum possible, because the entire tumor is sampled, and laboratory costs can be measured as the number of paraffin blocks. What we can address by modeling is increasing systematically the number of blocks used by the DAC strategy (ie., its cost) and computing, for different values of tumor ITH density, the mean performance of ITH detection. The results are shown in Table 1, where the parameters for the simulations are now L=27, C=1, N=50 and M=5. The DAC cost is normalized to the TTS cost (which in this case coincides with the tumor size, ie., for a 2D square, *L* ^2^ = 729). One can see from Table 1 that DAC strategy performed very well in detecting ITH, as compared to TTS but, with a much smaller cost; for instance, even for the most difficult situation of detecting a very low ITH density equal to 5% of the tumor (second row in Table 1) the mean DAC performance is equal to 94% (8th column in Table 1) but only demanding 3.5% of the TTS cost (8th column). Other situations are given in Table 1. This finding is of extraordinary importance for laboratory work, and adds further advantages for DAC in comparison to RP, balancing a higher reliability to a lower cost.

**Table 1.** Estimation for laboratory costs of DAC strategy (ITH types=1).

	DAC cost normalized to TTS cost = 0.5%	1.0%	1.5%	2.0%	2.5%	3.0%	3.5%
ITH density = 5%	**Mean** **performance** **in ITH** **detection =** **32%**	**52%**	**67%**	**77%**	**87%**	**90%**	**94%**
10%	**50%**	**78%**	**89%**	**95%**	**98%**	**100%**	**100%**
15%	**69%**	**90%**	**97%**	**99%**	**100%**	**100%**	**100%**
20%	**81%**	**95%**	**99%**	**100%**	**100%**	**100%**	**100%**
25%	**86%**	**98%**	**100%**	**100%**	**100%**	**100%**	**100%**

## Conclusions, take-home message

ITH is ubiquitous in CCRCCs, and current protocols for tumor sampling do not guarantee its detection, which is crucial for modern medicine as ITH affects patient prognosis and treatment. This new clinical need cannot be fully assessed using old pathological approaches as any solution to this problem must be cost-effective. This paper, resulting from the interaction between a practical pathologist and a basic researcher, suggests that a simple DAC strategy for tumor sampling is a very efficient approach for ITH detection, outperforming RP sampling at the same cost, and performing similar to the idealized strategy of TTS but with a (much) smaller cost (eg., for tumors with ITH densities of a 5%, DAC performs 94% equally well than TTS but with a 3.5% its cost). This issue is really critical when sampling large-sized tumors and makes it relevant for economical sustainability of health systems. We expect that the DAC strategy (validated here with synthetic data) might provide new light for a shift in the paradigm about how pathologists should sample tumors to achieve efficient ITH detection.
