# Supplementary material for: A divide-and-conquer strategy in tumor sampling enhances detection of intratumor heterogeneity in routine pathology: A modeling approach in clear cell renal cell carcinoma
Source: F1000Res. 2016 Apr 25;5:385. Originally published 2016 Mar 22. [Version 2] doi: 10.12688/f1000research.8196.2 (PMC4830216; doi:10.12688/f1000research.8196.2)
Supplement: Peer review history

# Peer review: Referee response for version 1

**DOI:** 10.5256/f1000research.8816.r13017

**Published:** 2016-04-29

**Authors:** Mattia Barbareschi, Noemi Dessì

**Affiliations:** 1 Unit of Surgical Pathology, Department of Pathology, Santa Chiara Hospital, Trento, Italy

Research and diagnosis of human tumours is based upon the analysis of small samples of the neoplasms, which are collected by surgical pathologist during macroscopic examination of surgically resected tumours. The study of Lopez and Cortes proposes a new method for tumour sampling for histological and molecular analysis, which could be particularly useful for large tumours  (diameter > than 3 cm) with high intratumour heterogeneity (ITH).  Detecting ITH is indeed challenging especially for large tumours which cannot be examined in toto and this  issue may be a limitation for research and therapy. The method suggested by Lopez and Cortes is essentially based upon a new multiple sampling strategy that could overcome some of the limitations of current sampling protocols. Their assumption is that usual sampling protocols (examining sampling roughly 1 large tissue fragment every centimeter of tumour size), do not allow a reliable evaluation of  ITH, which could be ideally achieved with a total tumour sampling strategy (TTS) which is however not sustainable by routine diagnostic laboratories. Lopez and Cortes suggest that performing multiple smaller samples for each tumour collected from randomly selected multiple tumour areas would more effectively detect ITH. Lopez and Cortes defined this strategy as “divide-and-conquer” (DAC) and  used a sophisticated mathematical modelling approach to demonstrate that it largely outperforms standard protocols with similar laboratory costs. The data of Lopez and Cortes are based on synthetic data and suggest for  that DAC performs 94% in detecting ITH as compared with an idealized TTS strategy, but at a much lower cost.

The paper of Lopez and Cortes is very interesting and it is an incentive to consider new sampling strategies of human tumours and should prompt additional studies using real tumour samples to verify whether these synthetic data reflect real life and if this approach will allow a more accurate and relevant histopathological and molecular analysis.

We have read this submission. We believe that we have an appropriate level of expertise to confirm that it is of an acceptable scientific standard.

# Peer review: Referee response for version 1

**DOI:** 10.5256/f1000research.8816.r13021

**Published:** 2016-04-11

**Authors:** Samuel Johnson

**Affiliations:** 1 Centre for Complexity Science, Warwick Mathematics Institute, University of Warwick, Coventry, UK

This paper provides a very elegant solution to the problem of detecting intratumour heterogeneity. The authors show quite convincingly that, at least in their modelling framework, the proposed divide-and-conquer method can outperform the routing protocol, and even yield results close to the ideal, total sampling method at a small fraction of the cost. I hope this idea will be taken up by researchers who can test the divide-and-conquer strategy in the lab, and eventually provide a significant improvement in tumour diagnosis.

I have read this submission. I believe that I have an appropriate level of expertise to confirm that it is of an acceptable scientific standard.

# Peer review: Referee response for version 1

**DOI:** 10.5256/f1000research.8816.r13020

**Published:** 2016-04-06

**Authors:** Francisco Nogales

**Affiliations:** 1 Department of Pathology, San Cecilio University Hospital, University of Granada, Granada, Spain

In the field of Gynaecological Pathology, tumours are known for their heterogeneous histology which often bear a prognostic significance, either by unmasking areas of malignant histology in an otherwise benign tumour of by evaluating multiple areas in tumour grading. To illustrate this, I would choose two particular tumours where multiple small tissue blocks, as proposed in the article, would be of help. An example involving the first situation would be mucinous tumours of the ovary, while the second one will be immature ovarian teratoma, where the semiquantitave evaluatiom of the number of developmentally immature neural and endodermal areas provides the histological grade necessary for managing the neoplasm.

This simple technique may be helpful with a lower cost in both working hours and laboratory material

I have read this submission. I believe that I have an appropriate level of expertise to confirm that it is of an acceptable scientific standard.

# Peer review: Referee response for version 1

**DOI:** 10.5256/f1000research.8816.r13018

**Published:** 2016-04-05

**Authors:** Nicola Ancona

**Affiliations:** 1 Institute of Intelligent Systems for Automation, National Research Council, Bari, Italy

The authors address the challenging problem of detecting of intratumor heterogeneity (ITH),  an important question in routine pathology with relevant impact on patient treatment and prognosis. Nowadays, pathologists follow standard protocols although current practice seem to be insufficient for detecting intratumor heterogeneity with high reliability. To assess ITH with high precision, the authors proposed a divide and conquer strategy, a very simple approach consisting in sampling many small-size pieces along the tumor. The proposed strategy has two advantages: a) increases the performances of standard protocols for detecting ITH and b) reducing the laboratory costs. To assess the performances of the proposed strategy, the authors designed an interesting statistical model and performed an accurate simulation.

The paper is well written and fit well the spirit of the journal. I have only one minor remark. The procedure adopted for detecting ITH sites in the simulation is not clear. Is this a random selection? Please add one sentence to make clearer this aspect. Finally, I found the paper extremely interesting, with high impact and relevance on current patient prognosis and treatment.

I have read this submission. I believe that I have an appropriate level of expertise to confirm that it is of an acceptable scientific standard.

# Response

**Published:** 2016-04-12

**Authors:** Jesus Cortes

**Affiliations:** Biocruces Health Research Institute, Spain

We thank the reviewer for making us clarify this. Yes, we use a random sampling as a first stage solution to this problem. We added a paragraph at the end of the section "Modeling approach" clarifying this.

We also added two new sentences for making the manuscript more clear; a new sentence in the "Introduction" briefly explaining what a divide and conquer strategy means and another sentence in the section "The problem" indicating why references (Lopez JI *et al.*  BJU Int 2012) and (Lopez et al. APMIS 2013) show very concerning data.
